# Polymer-Based Systems for Controlled Release and Targeting of Drugs

**DOI:** 10.3390/polym11122066

**Published:** 2019-12-11

**Authors:** Gaetano Giammona, Emanuela Fabiola Craparo

**Affiliations:** Laboratory of Biocompatible Polymers, Department of Biological, Chemical and Pharmaceutical Sciences and Technologies (STEBICEF), University of Palermo, Via Archirafi, 32-90123 Palermo, Italy

The current need to find new advanced approaches to carry biologically active substances (conventional organic drugs, peptides, proteins (such as antibodies), and nucleic acid-based drugs (NABDs such as siRNA and miRNA)) in the body fluids, to realize targeted therapies and even personalized ones, goes hand in hand with research on the performance of new materials to better realize appropriate drug vectors [1].

Polymeric materials can be designed and manufactured to obtain delivery systems with the appropriate characteristics in terms of drug release and performance [2]. For use in human applications, the polymer must primarily be biocompatible and non-toxic, and then functionalizable to give the appropriate structural and functional characteristics, such as to make it easily workable, processed, and engineered to obtain the desired system, and to be applied in drug delivery and targeting and/or in diagnosis of diseases. 

The further possibility of decorating the surface of these polymeric systems (due to the characteristics of the material that constitutes the matrix) with ligands capable of interacting specifically with membrane receptors on cells represents a unique advantage for obtaining targeted drug release to a specific organ, tissue, or cell type [3,4,5,6,7].

In this issue, some current examples of design and production of polymeric materials, as well as of searching strategies to modify existing ones, for the making of innovative systems for drug delivery and/or regenerative medicine are collected.

In particular, polymeric systems from nanoscale (micelles [8,9], nanoparticles [10,11]) to microscale structures (microparticles [12,13]), and to macrodevices (hydrogels [14] and films [15]) were produced. All the described systems were designed for the controlled and targeted release of conventional or biological drugs, such as paclitaxel [10], or siRNA [11] in the treatment of diseases such as cancer [8] and buccal and skin infections [15,16] by the systemic or local administration route [17]. The starting polymeric materials were chosen from hydrophilic polysaccharides [11,16] to hydrophobic polyesters [9,14], obtaining blended materials or copolymers, which were used to obtain drug delivery systems by using techniques such as microfluidics or hot punching [12,13].

Polymeric porous microparticles are currently emerging due to their potential for various applications, such as floating drug delivery systems and inhaled formulations. Amoyav and coworkers described the preparation of porous microspheres (MPs) starting from poly(lactic-co-glycolic) acid (PLGA) and poly(d,l-lactide) (PLA), with varying sizes and morphologies, by a simple flow-focusing microfluidic device [13].

Characterization of obtained systems to predict the in vivo fate is a fundamental aspect for researchers. Abid and coworkers described the production of microdevices, starting from different polyesters (i.e., poly-ɛ-caprolactone (PCL) and poly(lactic-co-glycolic acid) (PLGA)) by hot punching, and their characterization in terms of mucoadhesion with an ex vivo retention model and degradation studies in the presence of pancreatic enzymes [12].

Genetic material represents the new therapeutic approach to managing diseases. Sardo and coworkers described the production of redox-responsive siRNA-loaded systems for magnetofection [11]. In particular, siRNA-loaded magnetoplexes were able to release siRNA in a redox-triggered manner due to intracellular glutathione (GSH) mediated reduction of disulphide bridges formed during the crosslinking process. In another paper, the characterization and optimization of PLA in in-situ forming hydrogels (that exhibited a sol-to-gel transition between room and body temperatures), composed of PEI/DNA multi-layered micelles, for local gene delivery systems was described [14]. The investigation of their degradation profiles and chemical analysis indicated the faster acidic degradation and stepwise degradation process of these micelle–hydrogel systems.

Temperature-responsive behavior, as well as the capability to respond to pH or a reducing environment, is achieved for systems ranging from nano- to microdevices to control the release of drugs. Zhang and coworkers described the realization of nanoparticles starting from a temperature-responsive PEGylated polyaspartamide derivative, which were used to carry paclitaxel, showing suitable characteristics that make it a promising drug delivery system [10]. In another paper, biodegradable polymeric micelles based on a polyurethane–polyethylene glycol copolymer with disulfide bonds in the main chain (PEG–PU(SS)–PEG) were produced [8]. These systems were able to enable quick release of entrapped doxorubicin under intracellular reducing conditions. Zhai and coworkers described the production of pH-sensitive doxorubicin prodrug (mPEG–PCL–Imi–DOX) forming micelles that were responsive to the acidic tissular or intra-tumor microenvironment [9]. In particular, the macromolecular prodrug was synthesized by chemical conjugation of doxorubicin to the polymer via acid-cleavable imine bonds, and DOX release from the prodrug micelles was pH-responsive and able to be accelerated with a decrease of pH.

Topical administration on the skin or buccal mucosa represents a valid alternative to increased drug efficacy and reduced side effects related to systemic or oral administration of drugs. Marto and coworkers described a new approach to treat superficial skin infections by topical application of antibiotics, such as minocycline hydrochloride, formulated in a novel starch-based Pickering emulsion [16]. Junmahasathien and coworkers described the realization of pectin films, loaded with metronidazole, for the treatment of periodontal disease [15]. The preliminary results showed that low methoxyl pectin film containing glycerin and metronidazole could be potentially considered as a promising clinical tool for drug delivery via an intra-periodontal pocket to target an oral disease that is associated with polymicrobial infection.
